# Correction to “Perceptions of nursing workloads and contributing factors, and their impact on implicit care rationing: A Queensland, Australia study”

**DOI:** 10.1155/jonm/9834257

**Published:** 2026-05-28

D. G. Hegney, C. S. Rees, R. Osseiran‐Moisson, et al., “Perceptions of nursing workloads and contributing factors, and their impact on implicit care rationing: A Queensland, Australia study,” *Journal of Nursing Management*, 27, no. 2 (2019): 371–380, https://doi.org/10.1111/jonm.12693.

In the article, there is an error in the sixth paragraph of the discussion. Specifically:

• “This finding was seen in our previous studies (insert references after peer review).”

Should read:

• “This finding was seen in our previous study [1].”

We apologize for this error.
